# Medical Charity Question

**Published:** 1873-11

**Authors:** 


The Medical Charity Question.—The medical charity question is still open for discussion, and we are willing that it shall be so long as any new ideas are elicited or new suggestions made. In another column we have letters bearing upon the subject—one in particular referring to the relation of medical charity to the clerical profession—which deserves attention. Our correspondent, who seems to be more than usually favored with ministerial patronage, is inclined to look at the matter in the practical light of a man of business who is seeking some explanation for acts which have been sanctioned by a time-honored custom, and which he in common with others of the profession are expected to perform whenever called upon.

As far as this particular question of gratuitous services is concerned it resolves itself into one of courtesy, of sympathy for a class of gentlemen whose lives are spent in self-sacrifice for the good of others. The custom, doubtless, dates as far back as the time of the Apostles, and is, in the main, a good one; one which does honor to our hearts and consciences. There may be exceptions to the general application of the accepted rule, but these are certainly very few indeed, not enough in themselves to have any particular weight as militating against the general practice.

As a rule, clergymen have small incomes, relatively much smaller than physicians of correspondiug standing in their profession. They have a better start in life than physicians, but they are proportionately longer at a standstill when they arrive at a point of barely living.

Again: they do not ask our services for nothing, but that should not relieve us of our self-imposed obligations. If our correspondent finds his clerical charity a burden, he has only himself to blame for forcing himself to do what he believes to be wrong. We are understood now to speak on general principles, and from such a standpoint to sanction the custom. If a physician be very poor, and the clergyman very rich, the case is altered, in a pecuniary sense at least, and charges should be made. We do not think that there would be any objection to paying bills under these circumstances—no more objection than there should be for charging a fee.

Our correspondent has never heard of a clergyman refusing a fee. If a physician refuses a fee when he can get it, he can certainly learn a useful lesson from the minister. We should like to see what would be the result if our correspondent should be offered a fee by one of his five clerical patients. If he should refuse, it would not be because he thought it wrong to do so.

We bestow our charities upon the ministry for two very good reasons, which we cannot conscientiously offset by a merely pecuniary consideration. First, they are gentlemen whose pay, in a general sense, bears no comparison to their work, making them as a class relatively poor. Secondly, we should take a particular gratification in extending professional aid to such active and efficient co-workers in the cause of humanity and suffering.

